# Neutrophils, Fast and Strong

**DOI:** 10.3390/biomedicines10082040

**Published:** 2022-08-21

**Authors:** Galina F. Sud’ina

**Affiliations:** Belozersky Institute of Physico-Chemical Biology, Lomonosov Moscow State University, Moscow 119234, Russia; sudina@genebee.msu.ru; Tel.: +7-495-939-3174

The history of medicine is also the history of our understanding of the role of neutrophils in protecting our bodies. Ilya I. Mechnikov discovered that phagocytes and phagocytosis are the basis of natural cellular immunity, and macrophages and microphages, later renamed neutrophils, are the first line of defense in the host’s response to damage, infection, and tissue repair [1]. Neutrophils are fast and strong, and protect the body in all pathologies [2,3,4,5,6]. In this context, the study of the functions of neutrophils in health and disease is of great interest and importance.

## 1. Original Research Articles: New Approaches for Balancing Neutrophil Responses

Neutrophils are the first to enter the site of infection. A balanced neutrophil response is essential for effective pathogen destruction with minimal tissue damage. In the original research article of El-Benna and colleagues [7], it was found that the prolyl-isomerase Pin1 controls key neutrophil inflammatory functions. Thus, inhibitors of Pin1 impair proinflammatory functions of neutrophils. 

The functional activity of neutrophils is very strongly impaired in hematological malignancies, predisposing to tumor growth and resistance to chemotherapy. It has been established that the immunomodulatory drugs lenalidomide and pomalidomide improve the functional activity of neutrophils [8]. 

To quantify neutrophil responsiveness in normal and inflammatory conditions, a multiparametric analysis of neutrophil reactivity with respect to depolarization, alkalinization, and cell size change was introduced, and provided an approach to simultaneously quantify these changes using multiparametric flow cytometry [9]. 

Neutrophils are actively involved in inflammatory processes in Alpha-1 antitrypsin (AAT) deficiency (AATD). Dysregulation of neutrophil degranulation has been found to be an important feature in the pathogenesis of AATD [10]; this study supports approaches to the therapeutic use of AAT augmentation therapy to balance the neutrophil response. 

Neutrophils are the first line of defense against cancer cells that metastasize to the bloodstream. Bremer and colleagues [11] established that Galectin-9 sensitizes carcinoma cells to immune recognition and activates neutrophils, which leads to trogocytosis of cancer cells; neutrophil-mediated anti-cancer immunity is greatly enhanced by galectin-9.

It has been shown that neutrophils accumulate in the intraluminal thrombus and contribute to the progression of abdominal aortic aneurysm (AAA) [12]. Activated neutrophils form neutrophil extracellular traps (NETs) [13]. In a clinical study in AAA patients, it was found that neutrophil activation and NET parameters are higher in the blood of AAA patients, and the stimulating role of oxidized low-density lipoproteins (oxLDL) in the formation of NETs was shown [14].

Mechanisms of adhesion and secretion in neutrophils play an important role in neutrophilic inflammation, and hyaluronic acid (HA) mediates the recruitment of neutrophils into tissues [15]. The study of the 4-methylumbilliferon (4-MU), an inhibitor of HA synthesis, on the secretory processes in neutrophils revealed anti-inflammatory effects of 4-MU; 4-MU reduced secretion of granule proteins, including pro-inflammatory components [16], which may be the basis for therapeutic use of 4-MU. 

The clinical significance of blood neutrophils is especially pronounced in SARS-CoV-2-infection-induced coronavirus disease (COVID-19). Neutrophils infected with SARS-CoV-2 had lower levels of MPO release and had a direct effect on the number of peripheral blood lymphocytes, reducing their number [17]. 

Neutrophils form NETs that are toxic for microorganisms [18]. Laminin potentiated NET release increased the *Leishmania* capacity to induce NETs, and NET-rich supernatants were toxic for *Leishmania* [19].

There are differences in neutrophil characteristics in sepsis, trauma, and control patients. Thorough analysis was performed by Haitjema and colleagues, and increased neutrophil size for both sepsis and trauma patients was reported [20]. 

## 2. Review Articles: Neutrophils in Inflammatory Diseases

Neutrophils and molecules of neutrophilic origin play a critical role in cardiovascular diseases. Neutrophil-derived factors were analyzed in detail as potential biomarkers of neutrophilic origin in abdominal aortic aneurysms (AAA) [21]. A combination of clinical and experimental biomarkers may improve the diagnosis and prognosis of AAA. 

Neutrophil-driven NETs are associated with many diseases. Youichi Ogawa et al. presented a review on the involvement of NETs in the mechanisms of dermatological diseases associated with neutrophils [22]. Based on the involvement of NETs in many neutrophil-related skin disorders, including psoriasis and pustular dermatosis, NETs inhibition is seen as a promising therapeutic option.

In the pathophysiology of COVID-19, NETs have also been described as potential biomarkers of COVID-19 prognosis [23]. NETs enhance the systemic inflammatory and thrombotic response, increasing the severity of COVID-19.

The function of neutrophils in inflammatory processes and disease progression in osteoarthritis is reviewed by Akkouch and colleagues [24]. Therapeutic approaches are mainly focused on mitigating the destructive activity of neutrophils. 

The inflammatory/anti-inflammatory and various double-edged paradoxical activities of neutrophils have been reviewed, and the role of neutrophils in causing severe complications, including immune dysregulation in COVID-19, has been discussed [25]. 

Neutrophils are abundant leukocytes in the circulation, and have activities as in infection processes, as well as in sterile inflammatory processes. Neutrophils are fast and strong, and all described processes are important for balancing neutrophil responses. 

## Data Availability

Not applicable.
